# Characterization of Zinc and Cadmium Hyperaccumulation in Three *Noccaea* (Brassicaceae) Populations from Non-metalliferous Sites in the Eastern Pyrenees

**DOI:** 10.3389/fpls.2016.00128

**Published:** 2016-02-09

**Authors:** Soledad Martos, Berta Gallego, Llorenç Sáez, Javier López-Alvarado, Catalina Cabot, Charlotte Poschenrieder

**Affiliations:** ^1^Plant Physiology Laboratory, Bioscience Faculty, Universitat Autònoma de BarcelonaBarcelona, Spain; ^2^Botany Laboratory, Bioscience Faculty, Universitat Autònoma de BarcelonaBarcelona, Spain; ^3^Biology Department, Universitat de les Illes BalearsPalma de Mallorca, Spain

**Keywords:** *Noccaea brachypetala*, genetic markers, morphometric markers, Zn hyperaccumulator, Cd hyperaccumulator, non-metalliferous soil

## Abstract

The Southern slope of the Pyrenees is the meridional limit for the distribution of several *Noccaea* populations. However, the systematic description of these populations and their hyperaccumulation mechanisms are not well established. Morphological and genetic analysis (ITS and 3 chloroplast regions) were used to identify *Noccaea* populations localized on non-metallicolous soils during a survey in the Catalonian Pyrenees. Cd and Zn concentrations were analyzed in soils and plants both sampled in the field and grown hydroponically. The expression of selected metal transporter genes was assessed by quantitative PCR. The populations were identified as *Noccaea brachypetala* (Jord.) F.K. Mey by conspicuous morphological traits. Principal component analysis provided a clear separation among *N. brachypetala, Noccaea caerulescens* J. Presl & C. Presl and *Noccaea occitanica* (Jord.) F.K. Mey., three *Noccaea* species reported in the Pyrenees. Contrastingly, ITS and cpDNA analyses were unable to clearly differentiate these taxa. Differences in the expression of the metal transporter genes *HMA3, HMA4*, and *MTP1* between *N. caerulescens* and *N. brachypetala*, and those amongst the *N. brachypetala* populations suggest differences in the strategies for handling enhanced Cd and Zn availability. This is the first report demonstrating Cd and Zn hyperaccumulation by *N. brachypetala* both in the field and in hydroponics. This comprehensive study based on taxonomic, molecular, and physiological data allows both the correct identification of this species and the characterization of population differences in hyperaccumulation and tolerance of Zn and Cd.

## Introduction

During the last decade metal hyperaccumulating plants have attracted much research interest because of their potential use in clean-up of metal contaminated soils. While the direct use of hyperaccumulators is hampered by the usually low biomass of most of these species, the identification of the mechanisms underlying hyperaccumulation can provide the genetic tools for engineering more efficient plants for phytoremediation (Prasad and Freitas, 2003; Krämer, 2005a).

*Noccaea caerulescens* is a model plant for studying Zn and Cd hyperaccumulation and huge amount of information on this species is available, especially on the Ganges and Prayon ecotypes (Assunção et al., 2003b; Milner and Kochian, 2008; Halimaa et al., 2014). Other *Noccaea* species have obtained less attention, especially in mechanistic studies.

The number of species within the genus *Noccaea* (Brassicaceae) has been estimated in 154 (Al-Shebaz, 2014). Many hyperaccumulate Zn (35 species), some of them accumulate Ni (15 species). *Noccaea caerulescens* and the closely related *N. praecox* (Vázquez et al., 1992; Vogel-Mikuš et al., 2005) have been described as Cd hyperaccumulators. Moreover, analysis of herbarium material has revealed hyperaccumulation of Zn and Cd in *N. brachypetala* (Reeves et al., 2001).

*Noccaea caerulescens* is one of the most variable and taxonomically difficult species of the genus *Noccaea* (Koch and German, 2013). These authors remark that the adequate taxonomical category of this species has been largely ignored by an important part of the European studies and its putative distribution and that of similar species is uncertain. The presence of *N. caerulescens* has been described both on metalliferous and on non-metalliferous soils with a widespread, but uncommon, distribution in Western and Central Europe (Clapham and Akeroyd, 1993). France with nearly 80 listed sites for *N. caerulescens* has probably the best-known distribution of this species in one country (Reeves et al., 2001). Most of these sites are located in Southwest France, and just two populations are indicated by the authors in the Northern Pyrenees. Spain is the southern limit of this species and three Spanish databases (see Materials and Methods) locate all the known populations in the northern part of the Peninsula (from the west to east: Galicia, Cantabria, Basque Country, Aragon and Catalonia). All the eastern Spanish locations are in the Pyrenees.

According to Koch and German (2013) the limit between *N. caerulescens* and *N. brachypetala* is unclear, and some specimens from central and eastern France are either considered as synonyms or as subspecies. Nevertheless, Al-Shebaz (2014) recognizes both taxa as separate species. Meyer (1973) accepted three subspecies for *N. brachypetala* (subsp. *brachypetala*, subsp. *tatrensis*, and subsp. *huteri*), but the concept should to be revised when considering populations from Central France and Spain (Koch and German, 2013).

In this context, the first aim of this study was to contribute to accurately identify the populations of *Noccaea* from the eastern Pyrenees based on both morphological features and genetic data. The second aim was to elucidate the ability of the three non-metallicolous Pyrenean populations to accumulate Cd and Zn and to characterize the underlying mechanisms, using as a reference the well-characterized *N. caerulescens* (Ganges). For this purpose, both field and hydroponic studies were performed. Plant growth and the differential expressions of key metal transporters under Cd and Zn exposure were compared among *N. caerulescens* Ganges and the Pyrenean populations to link metal tolerance to the molecular bases of metal transport and compartmentation. In this regard, our study addresses the lack of combined taxonomical and physiological studies highlighted by Koch and German (2013).

Revealing the molecular basis of metal transporters in *Noccaea* populations of the Pyrenees can be of the special interest related to the proposals underlined in different phytoremediation reviews (Krämer, 2005a; Pilon-Smits, 2005). In the last decade the use of genetically modified plants for the extraction of metals and metalloids from the soil has been one of the biotechnological achievements. The main approaches to obtain these cleaning-up plants are the implementation of metal transporters, improved production of enzymes of sulfur metabolism and production of metal-detoxifying chelators-metallothioneins and phytochelatins (Kotrba et al., 2009). Moreover, Bhargava et al. (2012) pointed out that knowing the genetics and biochemistry of metal uptake, transport and storage in hyperaccumulator plants will lead to develop transgenic plants with improved phytoremediation capability. Although there is progress in this field and some plants have been modified with metal transporter genes (Antosiewicz et al., 2014; Iqbal et al., 2015), the achievement of an engineered plant with hyperaccumulation traits ready to be used in phytoextraction technology is still a pending challenge.

## Materials and methods

### Plant and soil sampling

In order to localize *Noccaea* species in the southern slope of the eastern Pyrenees a survey based on the information of different databases was performed: (1) Anthos program from the Spanish Royal Botanical Garden (http://www.anthos.es/), (2) Biodiversity Data Bank of Catalonia (BDBC; http://biodiver.bio.ub.es/biocat/) and (3) Flora Atlas of Aragon http://www.ipe.csic.es/floragon/).

**Figure 1:**
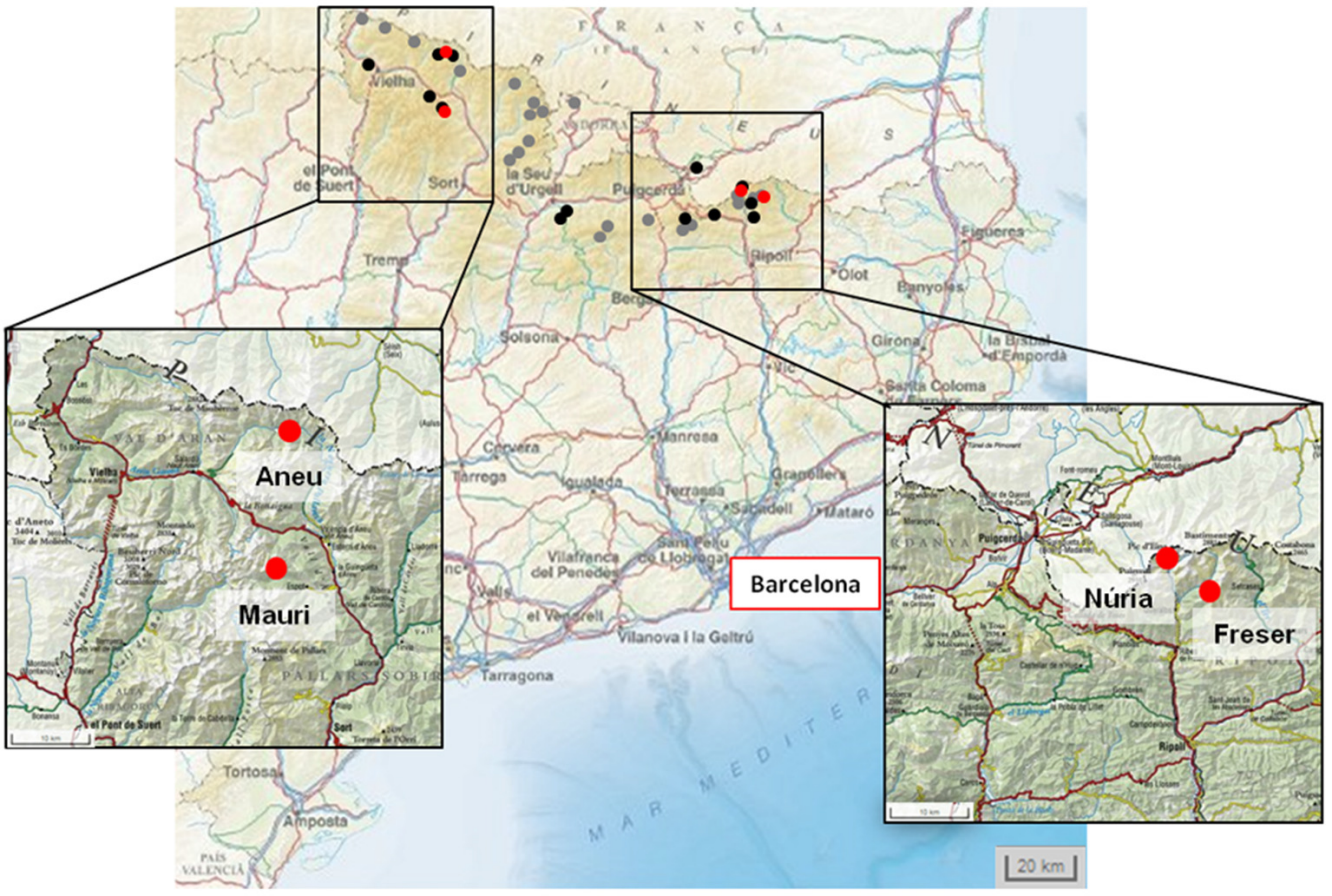
**Map of distribution of ***Noccaea*** species in the Catalonian Pyrenees**. Points indicate the 38 sites where Noccaea (*N. brachypetala, N. caerulescens*, and *N. occitanica*) are reported in the databases. Black and red points indicate the 16 sites visited during this survey; red points indicate sites where *N. brachypetala* populations were found; black points are sites where no *Noccaea* species could be found.

Using this database information, the distribution of *Noccaea* in the Catalonian Pyrenees was transferred on cartography marking all the localizations where the genus has been cited. A first sampling campaign was performed in spring 2011 when 16 out of the 38 described localizations were visited. During the sampling, it was observed that the original distribution of *Noccaea* is currently reduced and plants of this genus could not be found at most previously cited localizations. Finally, three *Noccaea* populations were located (Figure 1). Throughout the text these populations will be named as Aneu, Mauri, and Freser. In 2014 another population was detected 11 km north from Freser, in Núria. This population was only included in the morphometric and genetic analysis.

Fruit bearing plants from Aneu and Mauri populations were excavated together with adjacent soil and transferred to culture pots. After transport to the lab, potted plants were located into a growth chamber and regularly watered until seed ripening. The field-collected plants were used for morphological studies and mineral content analysis. As the Freser population was very small only some seeds were collected and no plants were removed for not disturbing this fragile population. At each site, soil close to the plants was collected in triplicate using an Edelman drill, universal type of 7 cm of diameter (Eijkelkamp, Giesbeek, Netherlands) at a depth of 10 cm.

### Morphological study

Positive identification of the three Pyrenean populations was based on morphological and anatomical observations of collected plants and herbarium specimens from two herbaria of the Botanical Institute of Barcelona (BC) (http://www.ibb.bcn-csic.es/en/documentacio/herbari/) and the University of Barcelona (BCN) (http://crai.ub.edu/es/conoce-el-crai/CeDocBiV). Representative herbarium material examined is listed in the Supplementary Material (Table S1). Morphological characters traditionally used in floristic or taxonomic treatments were examined (Bolòs and Vigo, 1990; Clapham and Akeroyd, 1993; Pujadas, 1993; Bolòs et al., 2005; Al-Shebaz, 2014). Features of gross morphology were studied under a Zeiss binocular stereoscopic microscope.

A total number of 41 specimens (13 for *N. caerulescens*, 17 for *N. brachypetala* and 11 for *N. occitanica*) were used for a morphometric multivariate analysis. Twelve quantitative and continuous characters were selected for their taxonomic discriminating value. The normality of each character was tested using the Shapiro-Wilks statistic (Shapiro and Wilk, 1965) and no transformation was needed for any of the characters. All morphometric analyses were computed using SPSS 15.0 (SPSS, 2006). The analysis based on a correlation matrix (PCA; Sneath and Sokal, 1973) was performed using individuals as OTUs (operational taxonomic units). PCA was used to reduce the overall variation of the 12 characters examined into 2 new uncorrelated components to represent morphological relationships among individual specimens. Data relating to glands were not included in the multivariate analysis as it was impossible to obtain micro-morphological data for all of the 41 specimens reviewed in the morphometric study.

### Genetic data

To establish the genetic affinities of the three Pyrenean populations to *Noccaea* species described in the region and to related taxa from other sources fresh leaves from the collected Pyrenean plants, dried caulinar leaves from the herbarium specimens, and seeds from our personal collection or purchased from a commercial supplier (B & T World Seeds, Aigues-Vives, France) were used. Detailed information on the corresponding geographical origin and, if applies, the herbarium collection is shown in Supplementary Material (Table S2) along with the gene bank accession numbers.

The total genomic DNA was extracted using the NucleoSpin® Plant II Kit (Macherey-Nagel GmbH & Co. KG, Düren, Germany) according to the manufacturer's instructions. Amplifications of four non-coding regions (ITS1-5.8S-ITS2, *trnL-trnF, rpl32-trnL*^(*UAG*)^, and *trnQ-5*′*rps16*) were conducted using a MJ Mini™ Gradient Thermal Cycler (Bio-Rad Laboratories, California, USA) in a 20 μl reaction with IQ™ supermix (Bio-Rad Laboratories, California, USA). The ITS region was amplified and sequenced using primers ITS1 and ITS4 (White et al., 1990) and the protocol of Likar et al. (2010). All sequences of primers are detailed in the Supplementary Material (Table S3). The *trnL-trnF* region was amplified and sequenced using the primers c and f (Taberlet et al., 1991). The PCR procedure started at 95°C for 4 min, followed by 35 cycles of denaturation at 93°C for 1 min, annealing at 58°C for 1 min, extension at 72°C for 2 min, and a final extension at 72°C for 10 min. The amplification and sequencing of the *rpl32-trnL*^(*UAG*)^ region was performed using the rpl32F and trnL^(UAG)^ as forward and reverse primers, respectively (Shaw et al., 2007). The last chloroplast region, *trnQ-5*′*rps16*, was amplified and sequenced using the trnQ^(UUG)^ as the forward and rpS16x1 as the reverse primer, respectively (Shaw et al., 2007). The last two regions were amplified by using the program “rp116” (Shaw et al., 2005). All PCR amplicons were checked on 1% agarose, TBE 0.5x gels stained by SYBR® safe. Previous to sequencing, PCR products were purified by enzyme digestion of exonuclease I and Antarctic phosphatase (New England Biolabs, Massachussetts, USA). Sequencing was performed at Bioarray, S.L. (Parque Científico y Empresarial de la UMH, Elche, Alicante, Spain) on a BigDye Terminators v3.1 Cycle Sequencing kit (Applied Biosystems, California, USA) and analyzed on an ABI 3130 Genetic Analyzer (Applied Biosystems, California, USA).

The sequences were edited and aligned using Bioedit Sequence Alignment Editor (version 7.0.7.0) (Hall, 1999); alignment was also checked visually by sequential pair wise comparison (Swofford and Olsen, 1990). Phylogenetic analyses were carried out using the Bayesian method and the software MrBayes 3.1.2 (Huelsenbeck and Ronquist, 2001; Ronquist and Huelsenbeck, 2003). The best-available model of molecular evolution required for Bayesian estimations of phylogeny was selected using the Akaike information criteria (AIC) and Bayesian information criterion (BIC), as implemented in the software jModeltest 0.1.1 (Guindon and Gascuel, 2003; Posada, 2008), which considers nucleotide substitution models that are currently implemented in MrBayes 3.1.2. The GTR model (Tavaré, 1986), with variable base frequencies, was assumed to follow a discrete gamma distribution and was selected as the best-fit model of nucleotide substitution for the cpDNA and ITS-cpDNA combined dataset. For the ITS alignment, the symmetrical model with equal base frequencies and rate variation among sites (SYM+G) was selected (Zharkikh, 1994). Bayesian inference analyses were initiated with random starting trees and were run for 10^6^ generations. Four Markov Chains were run using Markov Chain Monte Carlo (MCMC) principal sample trees. One tree out of every 100 generations was saved, which resulted in 10,000 sample trees. Data from the first 2500 generations were discarded as the “burn-in” period, until values had stabilized prior to the 2500th generation. All sequences are deposited in the EMBL/GenBank/DDBJ Nucleotide Sequence Databases.

### Analysis of field-collected soil and plant samples

Collected soils at each point were sieved through a 2-mm sieve to homogenize the samples and to remove roots. One gram of homogenized soils were mixed with MQ water and the pH of the resulting aqueous solutions were measured by a pHmeter micropH 2002 (Crison Instruments S.A., Barcelona, Spain). Cd, Co, Cu, Pb, and Zn in soils from the three sampled locations were extracted with *aqua regia* and analyzed by ICP-MS (Perkin Elmer Inc., ELAN 6000, MA, USA) (Bech et al., 2012). The certified reference material CRM 142 R Light Sandy Soil was included for quality control. Prior to the analysis of root and shoot metal concentrations the field-collected plants from Aneu and Mauri populations were carefully washed and processed as described below. The metal concentrations in plants from Freser were not analyzed as the original population was too small to be sampled.

### Hydroponic studies

To verify metal hyperaccumulation in the three Pyrenean populations identified in this study the well-characterized *N. caerulescens* J. Presl & C. Presl (ecotype Ganges) was hydroponically grown along with the Pyrenean populations. Germination of seeds of *N. caerulescens* (Ganges) (B&T World Seeds, Aigues-Vives, France) and field-collected seeds from the three Pyrenean populations was synchronized by treatment with 10^−5^ M gibberellin at 4°C for 4 days. After germination seedlings were first grown on vermiculite irrigated with 25% strength Hoagland solution (pH 5.5) consisting of 1 mM of Ca (NO_3_)_2_, 1.25 mM KNO_3_, 0.5 mM NH_4_H_2_PO_4_, 0.25 mM MgSO_4_, 50 μM KCl, 25 μM H_3_BO_3_, 2 μM MnSO_4_, 2 μM Zn SO_4_, 0.5 μM NH_4_Mo, 0.5 μM CuSO_4_, and 20 μM Fe-EDDHA. After 2 weeks the seedlings were individually transferred to plastic pots (100 ml capacity) filled with continuously aerated nutrient solution which was renewed weekly. When plants were 1-month old, 100 μM of ZnSO_4_ or 1.5 μM of CdCl_2_ were added to the solution and plants were grown for a further 4-week period. Fifteen plants per species or population and treatment were used in each experiment. The experiment was carried out in a growth chamber with day/night temperature of 25/18°C, photoperiod 12 h light/12 h darkness and PAR 135 μE m^−2^ s^−1^.

### Growth, photosynthesis, and mineral analysis

Growth was assessed by recording rosette and root dry weight after the plant tissues had been oven dried at 60°C. To reveal possible metal toxicity effects leaf chlorophyll concentrations (CCM-300 chlorophyll content Meter, Opti-Sciences, NH, USA) and chlorophyll fluorescence (Fv/Fm ratio) (JUNIOR-PAM, Heinz Walz GmbH, Germany) were measured. Both parameters were quantified as the average of three independent measurements on fully-expanded leaves. Non-destructive parameters (roots scanning, fluorescence and content of chlorophyll) were measured at the beginning of the treatment (*t* = 0) and every 2 weeks (*t* = 2nd and 4th week).

After 4 weeks of metal exposure, plants were separated into roots and shoots, carefully washed with distilled water and oven dried (60°C). The dry material from 10 plants per population and treatment was homogenized to fine powder followed by acid digestion (HNO_3:_ H_2_O_2_ 69%: 30%, 5:2 v/v) in a hot-block digestion system (SC154-54-Well Hot Block™, Environmental Express, SC, USA). The concentrations of the selected elements (Ca, K, Mg, P, S, and B, Cu, Fe, Mn, Mo, Zn) were determined by ICP-OES (Thermo Jarrell-Ash, model 61E Polyscan, England) (Bech et al., 2012). The certified material BCR 62 *Olea europaea* was included as a reference material for quality control. The data shown are means of three independently analyzed samples from the homogenized powder.

### Expression of metal transporter genes

The expressions of selected metal transporter genes were analyzed in plants treated with Cd or Zn in hydroponics for 1 week as described above. Then five plants from each Pyrenean population and from *N. caerulescens* (Ganges) were divided into roots and shoots. Parts from each population were collected together and then directly immersed into liquid nitrogen, homogenized to fine powder and stored at –80°C until use. Total RNA of each pool (around 100 mg) was extracted with TRIzol® Reagent (Invitrogen, Molecular Research Center Inc., OH, USA) according to the manufacturer's instructions. RNA extracted was quantified with Nanodrop 2000 (Thermo Scientific, DE, USA) and then adjusted to 1 μg. Quality of RNA was checked by the OD A_260_/A_280_ (1.8—2.0) and OD A_260_/A_230_ (>1.8). The synthesis of cDNA was developed in a 20 μl reaction volume with iScript™ cDNA Synthesis Kit (Bio-Rad, CA, USA). The cDNA (1:50) was used as a template for quantitative PCRs using iTaq™ Universal SYBR® Green Supermix (Bio-Rad, CA, USA) and reaction was developed on a CFX384 or a CFX 96 Real-Time System (Bio-Rad, CA, USA). The plates were edited by the software CFX manager version 3.1.

The effects of Zn and Cd on the expression of metal transporters *HMA3, HMA4*, and *MTP1* were studied. Previously a trial with the primers described for *N. caerulescens* was done by a common PCR in order to test if the metal transporters primers fitted to the Pyrenean populations. Amplicons were electrophoresed in a 1% agarose gel and band sizes of *N. caerulescens* were compared to those obtained for the Pyrenean populations. The amplicons of the four genotypes for the three markers were afterwards sequenced and then aligned. The band sizes for the three Pyrenean populations were similar to *N. caerulescens* and also the blast showed high similarity among them (Figure S1). This result indicates that the primers designed for *N. caerulescens* amplified for the same region and are optimal to be used for the Pyrenean populations.

The primers used for the expression of *HMA3, HMA4* and *MTP1* are detailed in the Supplementary Material (Table S3). The expression of target genes was normalized to the expression level of *Tubuline* gene. Treatment influence on relative gene expression was calculated by the ratio (Efficiency of the target gene) ${}^{\text{Δ}{\text{C}}_{\text{T}},\text{target}\left(\text{calibrator}-\mathrm{test}\right)}$/(Efficiency of the reference gene) ${}^{\text{Δ}{\text{C}}_{\text{T}},\text{reference}\left(\text{calibrator}-\text{test}\right)}$ (Pfaffl et al., 2002). The calculated amplification efficiencies were 90.6% (*HMA3*), 95.9% (*HMA4*), 99.5% (*MTP1*), and 93.2% (*Tub*). The results are means of three independent samples from the homogenized powder.

### Statistics

Continuous data were analyzed by the software Statistica 7.0 (Stat Soft, Inc. OK, USA). Normal distribution was checked and data not adjusting to normal distribution were transformed with logarithm and sinus corrections, before applying parametrical tests, ANOVAs followed by Tukey HSD.

## Results

### Localization and identification of pyrenean *Noccaea* populations

During the 2011 survey only three *Noccaea* populations were found in the Catalonian Pyrenees. From the west to the east, the populations are located close to Estany de Sant Maurici (42°57′80.70″ N, 10°08′98.4″ E; altitude 1950 m, called “Mauri”), in the Vall de l'Àneu (42°76′63.05″ N, 1°05′39.98″ E; altitude 1500 m; called “Aneu”) and in the Vall del Freser (42°38′20.78″ N, 2°21′55.15″ E; altitude 1940 m, called “Freser”) (Figure 1). Aneu and Mauri populations developed below trees on subalpine meadows, with each population constituted by more than 15 specimens. In contrast, at Freser only 2 plants growing on a boundary path were found. This population was not stable throughout the years, as confirmed by later visits to the area. At 2014, a small population was detected in Núria, 11 km north from Freser. This population was only included in the genetic study and morphometric analysis. In the available databases, *N. caerulescens* is classified as an occasional species and correspondingly it should be widespread in the Pyrenees; but this survey revealed that the current distribution of *Noccaea* in the Pyrenees is reduced compared to previous information.

**Figure 2:**
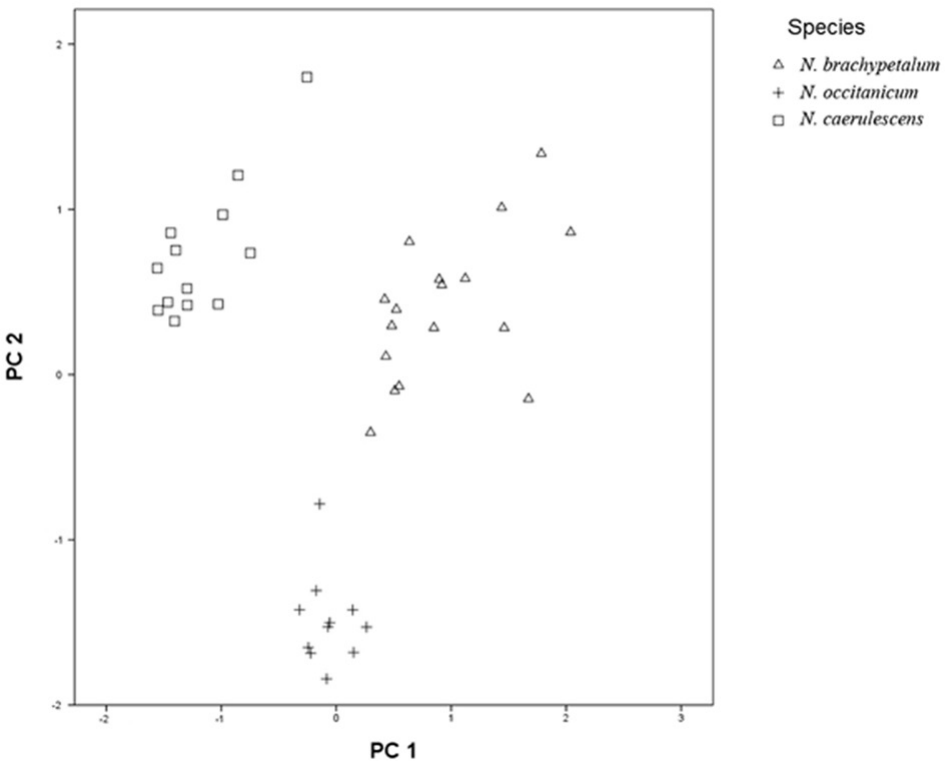
**Morphometric multivariate analysis based on a correlation matrix (PCA) of 12 taxonomic characters of the three ***Noccaea*** species recognized at the Pyrenees**.

Morphological studies (Table 1) comparing the sampled populations with herbarium specimens revealed that three *Noccaea* species are recognized in the Pyrenees: *N. brachypetala, N. caerulescens*, and *N. occitanica*. The morphometric multivariate analysis of 41 specimens identified three separated and independent groups providing integrity to these three species without an overlapping of the characters (Figure 2 and Table S4). This is consistent with the taxonomic treatments given by the “Flora Iberica” (Pujadas, 1993), the “Euro+Med PlantBase” (Markhold, 2011) and the synopsis of the genus *Noccaea* by Al-Shebaz (2014). The number and quality of the discriminating morphological characters found so far (Table 1A) and the lack of intermediate specimens linking the extreme morphotypes strongly supports the view that these three separate species are present in the Pyrenees. However, all specimens from the four populations we located in our survey were clearly identified as *N. brachypetala*, while we were unable to locate neither *N. caerulescens* nor *N. occitanica* in the studied area. Based on data obtained here together with those from previous publications a detailed morphological description of *N. brachypetala* is provided on the Table 1B.

**Table 1:** **Main morphological differences between ***Noccaea brachypetala, N. caerulescens***, and ***N. occitanica*** (A)**.

**(A)**	***N. brachypetala***	***N. caerulescens***	***N. occitanica***
Flower stalk length (cm)	13–55	5–40	4–19
Inflorescence in fruiting stage	Lax, rarely dense	Lax	Dense
Fruiting pedicels length (mm)	2–8	3–10	5–20
Sepals length (mm) and color	1–1.3	1.5–2.5	1.3–2
	Light green to yellowish-green	Light green, usually violet stained	Green and violet stained
Petals length (mm) and color	1–1.5, violet or white	2–4, white, sometimes pale rose or pale violet	1.8–3.2, white or pale rose
Stamen filament length (mm)	1.5–2	2–4.2	2.1–3.5
Anthers length (mm)	0.3–0.4	0.4–1	0.3–0.5
Style length (mm)	0.3–0.5	1–2 mm	0.7–1.7
	Included within the notch of the ripe fruit	Equaling or exceeding the notch of the ripe fruit	Included within the notch of the ripe fruit
Fruit size (mm) and shape	3.5–8 × 2–3.5	5–8 × 3–4	6–10 × 4–8
	Obcordate, not winged or narrowly winged basally, broadly winged apically	Oblong-cuneate to obcordate, narrowly winged basally, broadly winged apically	Broadly obcordate, broadly winged throughout its length
Seeds size (mm)	1.5–1.7 × 1–1.2	1.4–1.6 × 0.8–1.1	1.5–2.1 × 0.9–1.4
**(B)** ***Noccaea brachypetala*****:** Biennial or perennial; glabrous, glaucous. Flower stalks 13–55 cm long, erect, and usually unbranched. Basal leaves rosulate; petiole 5–20 mm; leaf blades oblanceolate, ovate, or elliptical, 5–24 × 2–9.5 mm, base cordate, margins entire, or denticulate, apex obtuse. Cauline leaves 3–20; blade ovate to lanceolate, 5–30 × 2–9 mm, base auriculate, margins entire, or denticulate, apex obtuse to acute. Racemes 3–22 cm, considerably elongated in fruit. Fruiting pedicels horizontal, straight, 2–8 mm. Sepals 1–1.3 mm, oblong, light green to yellowish-green; petals obovate to oblanceolate, 1–1.5 mm, white, erect. Stamens slightly tetradynamous, filaments 1.5–2 mm; anthers 0.3–0.4 mm, violet, green, or whitish. Style 0.3–0.5 mm included within the notch of the ripe fruit. Fruits 3.5–8 × 2–3.5 mm, obcordate, not winged or narrowly winged basally, broadly winged above (up to 2 mm width), with rounded apical lobes and a deep notch; base cuneate. Seeds 1.5–1.7 × 1–1.2 mm, slightly compressed, brown to reddish.			

*The morphological characters were analyzed by a binocular stereoscopic microscope. Detailed morphological description of N. brachypetala ***(B)****.

With regard to genetic data, the aligned matrix for ITS consisted of 21 sequences of 677 bp and 107 parsimony-informative characters. The cpDNA matrix consisted of 21 sequences of 1976 bp and 180 parsimony informative characters. The number of variable sites among *N. brachypetala, N. caerulescens* subsp. *caerulescens*, and *N. occitanica*, using the ITS-cpDNA combined dataset, ranges from 0 for *N. brachypetala* (populations Freser, Mauri, and Núria) and *N. occitanica*, to 3 for *N. brachypetala* (Freser, Mauri and Núria) and *N. caerulescens* subsp. *caerulescens* Prayon, and to 8 between *N. brachypetala* (Aneu) and the rest of the clade containing *N. brachypetala, N. caerulescens* subsp. *caerulescens*, and *N. occitanica*.

Bayesian analyses of ITS, cpDNA, and ITS-cpDNA combined datasets, provided similar topologies except for a significantly supported topological incongruence involving *N. rotundifolia* (L.) Moench, which was clustered in the ITS tree at the same clade as *N. brachypetala* and *N. caerulescens* but outside of this clade in cpDNA tree (trees not shown). Nevertheless, the matrices of ITS and cpDNA were combined due to the improvement in the phylogenetic signal and because the phylogenetic reconstruction of genus *Noccaea* is out of the aim of this work. Moreover, *N. rotundifolia* is not a key species in this study. The phylogenetic tree for ITS-cpDNA, with Bayesian posterior probabilities (PP), is shown in the Supplementary Material (Figure S2). Genbank accession numbers for the obtained sequences are generated as part of this study (Table S2).

### Metal concentrations in soils and *N. brachypetala* mother plants

**Figure 3:**
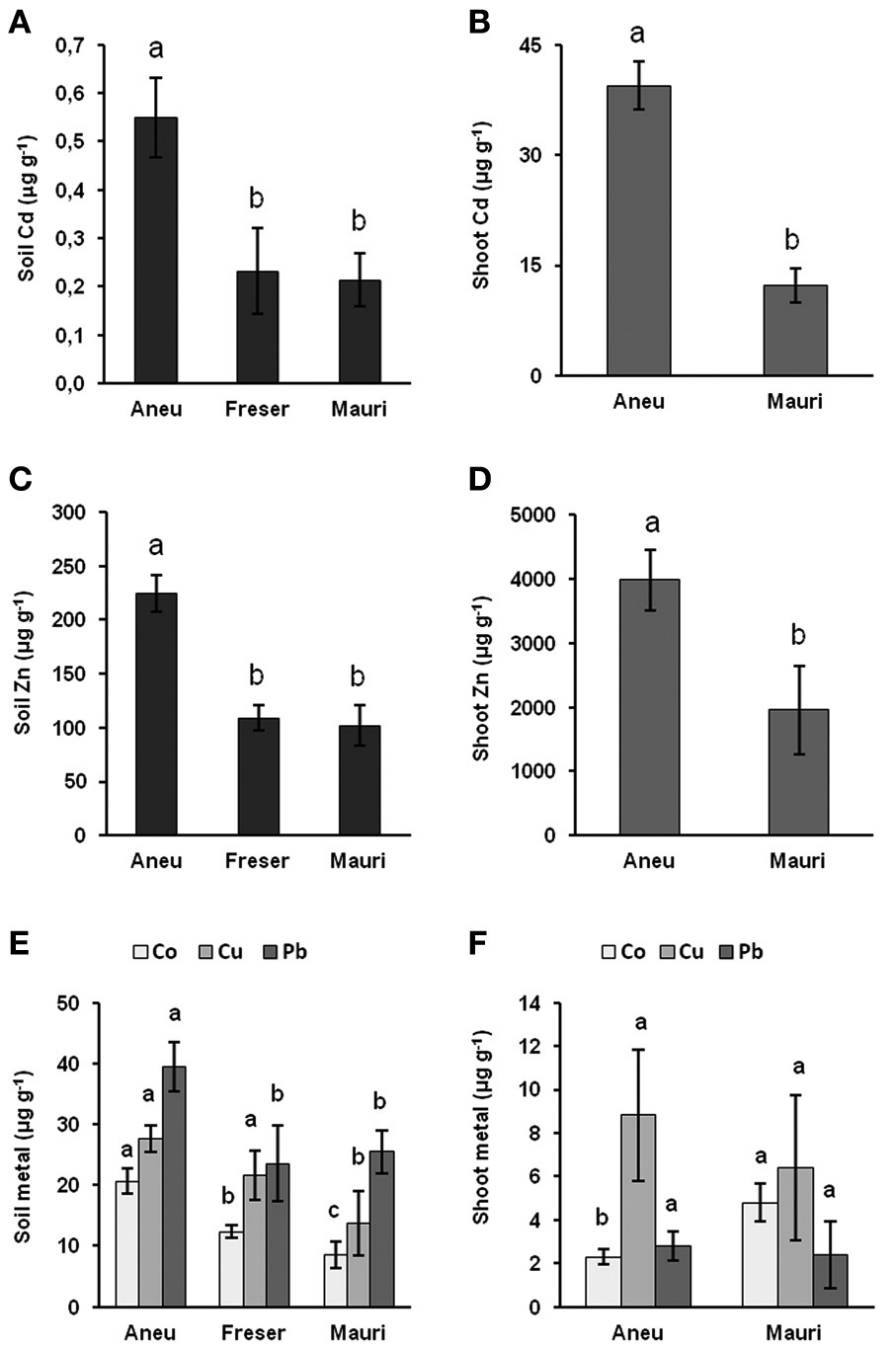
**Concentrations (μg g^−1^ dry weight) of Cd (A,B), Zn (C,D), and Co, Cu, and Pb (E,F) in soils (A,C,E) and ***N. brachypetala*** populations (B,D,F) sampled on the southern slope of the eastern Pyrenees**. Metal concentrations in field collected plants were only analyzed in Aneu and Mauri; Freser population was not collected because of the low number of individuals. Metal concentrations were analyzed by ICP-MS after *aqua regia* extraction.

**Figure 4:**
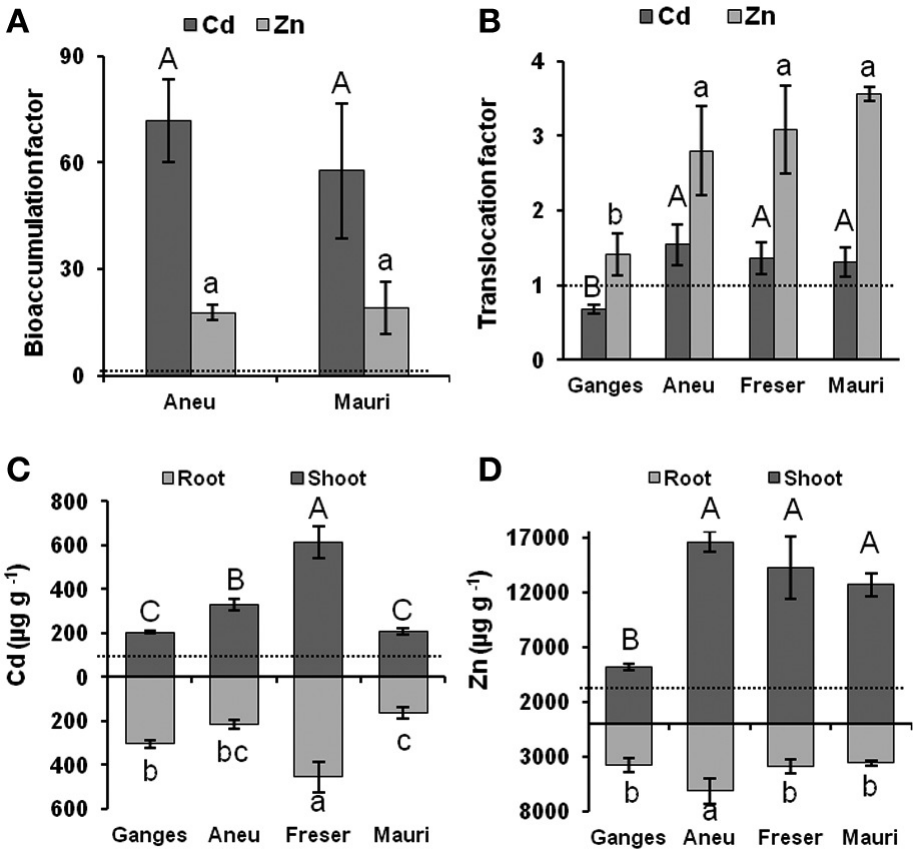
**Bioaccumulation factors (μg g^−1^ metal shoot/μg g^−1^ soil) in field sampled ***N. brachypetala*** populations (A); translocation factors (μg metal shoot/μg metal root) in ***N. brachypetala*** populations and ***N. caerulescens*** (Ganges) grown on hydroponics (B); Cd concentrations (μg g^−1^) in hydroponically-grown ***N. brachypetala*** populations and ***N. caerulescens*** (Ganges) (C); Zn concentrations (μg g^−1^) in hydroponically-grown ***N. brachypetala*** populations and ***N. caerulescens*** (Ganges) (D)**. Metal treatments on hydroponic plants were 1.5 μM CdCl_2_ and 100 μM ZnSO_4_, controls were without Cd or with 2 μM Zn. Plants were under treatment for 4 weeks. Error bars on columns in **(A,B)** are standard deviations based on three different ICP-MS analyses from a pool of five collected plants or five soil samples; columns with the same letters (capitals for Cd and lower case for Zn) are statistically not different (Tukey test; *p* < 0.05). Error bars on **(C)** and **(D)** are standard deviations based on three independent ICP analyses from a pool of 10 plants; columns with the same letters (capitals for shoots and lower case for roots) are statistically not different (Tukey test; *p* < 0.05) Dotted lines indicate threshold for hyperaccumulation.

Concentrations of Cd, Zn, Co, Cu, and Pb in the soil surface layer at Aneu, Freser, and Mauri (Figures 3A,B,E) were within the common range (Kabata-Pendias, 2011). No differences in the low soil metal concentrations between Mauri and Freser were observed, while soils from Aneu had higher contents. Soil pH values were 7.3, 5.9, and 5.2, for Aneu, Freser, and Mauri, respectively. Cd and Zn concentrations in the shoots of field-collected plants were extraordinarily high (Figures 3B,D). Aneu plants accumulated 70 times higher Cd and 18 time higher Zn concentrations than those found in the soil, and Mauri plants had 56 and 19 times higher Cd and Zn concentrations, respectively. In contrast, concentrations of Co, Cu, and Pb were lower in plants than in soils (Figures 3E,F). The bioaccumulation index relating the metal concentrations in the shoots to that of soils revealed the extreme ability of *N. brachypetala* to hyperaccumulate Zn and Cd (Figure 4A) from these unpolluted soils. However, no differences between the bioaccumulation factors of Aneu and Mauri were found due to the higher soil metal concentrations at Aneu.

### Growth, Zn, and Cd accumulation in hydroponics

The hydroponic experiments confirmed the high capacity of *N. brachypetala* to hyperaccumulate Zn and Cd and revealed the high Zn and Cd tolerance of these plants. All plants exhibited translocation factors greater than 1.0 (Figure 4B). No differences in the Zn and Cd translocation factors among the three *N. brachypetala* populations were found. Surprisingly, however, the *N. brachypetala* populations had higher translocation factors for Zn and Cd than *N. caerulescens* ecotype Ganges. *N. caerulescens* accumulated lower root and shoot Zn concentrations than *N. brachypetala* (Figure 4D). Shoot Cd concentrations were highest in *N. brachypetala* population Freser, followed by Aneu (Figure 4C). Mauri and Ganges had the lowest shoot Cd concentrations, but clearly above the hyperaccumulation threshold.

**Figure 5:**
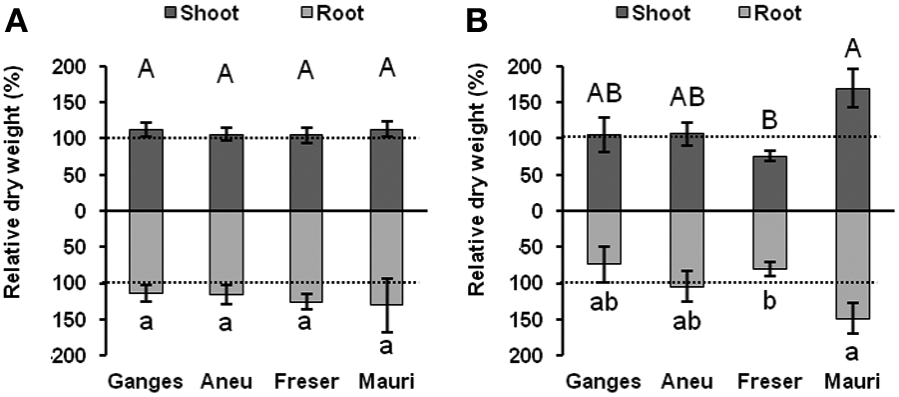
**Influence of exposure to 1.5 μM Cd (A) or 100 μM Zn (B) on plant dry biomass (% values relative to controls without Cd (A) or with 2 μM Zn (B) of different ***N. brachypetala*** populations and ***N. caerulescens*** (Ganges)**. Values are means ± SD (*n* = 10). Columns with the same letter are statistically not different (*p* < 0.05). Dotted lines mark 100%.

The hyperaccumulation of Cd did not affect the plant biomass in any population (Figure 5A). However, high Zn accumulation significantly reduced root and shoot growth in plants from Freser. In contrast, biomass production was stimulated by the high Zn supply in plants from the Mauri population (Figure 5B). Neither chlorophyll concentrations nor chlorophyll fluorescence parameters were affected by the metal treatments (data not shown).

### *HMA4, HMA3*, and *MTP1* expression

**Figure 6:**
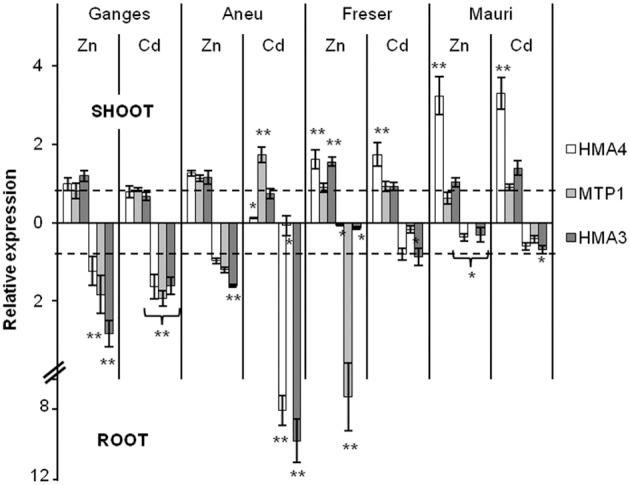
**Relative expression analysis of three metal transporters according to Pfaffl method**. The expression is expressed as the fold change compared to non-treated plants and normalized by tubulin gene. Plants were grown on a hydroponic system for 1 week under metal treatment of 1.5 μM CdCl_2_ or 100 μM ZnSO_4_, control plants were non-exposed to Cd or with 2 μM Zn. Shoot markers expression is represented on the top part of the graphic and root markers expression on the bottom part. Dotted lines mark similar expression (fold change around 1) for treated and control plants; double asterisk indicates over-expression (≥ 1.5) and single asterisk indicates down-regulation (≤ 0.5).

One week after the Zn or Cd treatment was initiated in the hydroponic study, *N. caerulescens* (Ganges) and the three *N. brachypetala* populations showed quite different patterns in the expression of metal transporter genes *HMA4, HMA3*, and *MTP1* (Figure 6). The Zn treatment enhanced the root expression especially of *MTP1* and *HMA3* in Ganges, but not in Mauri. In Aneu Zn exposure induced a slight enhancement of *HMA3* expression in roots. In contrast, in roots from Zn treated plants from Freser more than a seven-fold increase in the expression of *HMA3* was observed, while expressions of *HMA4* and *HMA3* decreased. However, *HMA4* expression showed a more than two-fold increase in shoots of Mauri. In Freser the Zn treatment caused a small, but statistically significant enhancement of the expression of *HMA4* and *HMA3* in shoots. In *N. caerulescens* Ganges Cd induced a close to two-fold increase in the root expression of *HMA4, HMA3* and *MTP1*. Cd-induced up-regulation of *HMA4* and *HMA3* was strongest in roots of Aneu achieving values close to eight-fold increase, while *MTP1* expression decreased in the roots and increased in the shoots. *MTP1* expression was also decreased in the roots of Cd-treated Freser and Mauri, while its shoot expression was substantially enhanced in plants from both populations (Figure 6).

## Discussion

According to literature and herbarium records *N. brachypetala, N. caerulescens*, and *N. occitanica* are more or less sympatric in the eastern Pyrenees. However, during our 3 year survey of the southern slope we only located 4 *Noccaea* populations. All of them could be identified as *N. brachypetala*. This identification is strongly supported by vegetative and reproductive characters (Table 1A, Table S4, and Figure 2) consistent with the taxonomical treatments done by Pujadas (1993), Markhold (2011), and Al-Shebaz (2014). Taking into account the morphometric values from 41 different *Noccaea* accessions (13 *N. caerulescens*, 11 *N. occitanicum*, and 17 *N. brachypetala*) the PCA analysis was able to clearly separate three groups corresponding to these taxa (Figure 2). According to the Eigenvalues the fruit length/fruit width ratio, the fruit width at apex, the stem width, and the style length/notch length ratio were the most determinant morphological parameters.

To support the morphological study and to clarify the relationships between *N. brachypetala, N. caerulescens* subsp. *caerulescens*, and *N. occitanica* on molecular grounds, a genetic analysis of four different regions was developed, using species-level phylogenetic markers. Some authors have used nuclear ribosomal DNA or chloroplast DNA to clarify the phylogenetic relationships within the Brassicaceae family (Franzke et al., 1998; Koch et al., 1998a,b; Bailey and Doyle, 1999; Koch and Mummenhoff, 2001; Koch and Al-Shehbaz, 2004; Koch et al., 2007), as well as in the genus *Thlaspi* (Mummenhoff and Koch, 1994; Zunk et al., 1996; Mummenhoff et al., 1997). However, taxonomies within Brassicaceae based on two or more datasets combining nuclear and chloroplast information, as presented here, are not generalized. Unfortunately, and in contrast to the cues from the morphological data, we were unable to detect enough phylogenetic signals to separate the *Noccaea* species by the genetic tools employed.

The poor genetic variation among the three *Noccaea* species could be explained by a recent or a rapid species radiation event, as denoted by the presence of short branches in the phylogenetic consensus tree (Figure S2). Moreover, incomplete lineage sorting or the presence of interspecific gene flow, mediated by weak genetic barriers, could be hampering the phylogenetic reconstruction as they act as homogenizing forces. The presence of these phenomena is demonstrated by the divergent positions where different accessions of *N. brachypetala* and *N. caerulescens* were recovered in the phylogenetic tree (Figure S2). The hypothesis of incomplete lineage sorting may be a better explanation since gene flow is unlikely in putatively inbreeding plants (Lombi et al., 2000; Besnard et al., 2009). Although, gene flow cannot be discarded especially taking into account that out-crossing rates in *N. caerulescens* can be much higher than originally thought (Koch et al., 1998b). Nonetheless, higher inbreeding coefficients have been observed in non-metallicolous than in metallicolous populations of *N. caerulescens* (Dubois et al., 2003).

Noteworthy, Mauri and Freser, non-metallicolous populations of *N. brachypetala*, were in the same clade as *N. caerulescens* Prayon, a metallicolous ecotype from heavily polluted soil. In contrast, Aneu from a soil with only slightly enhanced Cd and Zn concentrations was on a close, but different clade (Figure S2). Our data are in contrast with the high degree of genetic differentiation found in several studies for *N. caerulescens* (Koch et al., 1998b; Dubois et al., 2003; Basic and Besnard, 2006; Jiménez-Ambriz et al., 2007). However, these studies applied population genetics approach, using AFLP/isoenzymes and several individuals per population and, therefore they are not fully comparable. Future work should address this issue using more variable markers, such as AFLP or SSR, and focusing sampling effort on the closest relatives: *N. brachypetala, N. caerulescens* subsp. *caerulescens*, and *N. occitanica*.

In the Pyrenees the presence of these three *Noccaea* species is usually described in grasslands, meadows, prairies and occasionally in forests (Pujadas, 1993). According to the BDBC database, *N. caerulescens* is present in 28 UTM grids (10 × 10 km). Surprisingly, during our sampling campaign in 2011 searching 16 out of 38 described sites in databases for the occurrence of *Noccaea* and in further visits in 2012 and 2014, *N. brachypetala* was only located at Aneu, Mauri, Freser, and Núria, while neither *N. caerulescens* nor *N. occitania* were found. This becomes especially relevant when considering the fact that in the studied herbarium material, only four specimens are dated in the twenty-first century; while the rest of the records, 16, were between 40 and 140 years old. Therefore, based on the herbarium material collected data and on our experience in sampling in the Pyrenean range, it is likely that *Noccaea* is declining in this zone. Besides increasing tourism and urban activities and enhanced herbivore pressure due to cattle raising. This apparently recent decline of *Noccaea* could be caused by a low resilience to confront climate change, as this zone of the Pyrenees mountain range is on the southern limit of the distribution area of these species. There is clear evidence that the number of cold days per year has been declining during the last 40 years in the eastern Pyrenees (Morán-Tejeda et al., 2013) and this expectedly may have affected the reproductive fitness of *Noccaea* species which require vernalization for flowering.

The four populations identified in this study are located in cell N29E01 of the Global Terrestrial Network (GTN) of Forges countries in the Geochemical Atlas of Europe (http://weppi.gtk.fi/publ/foregsatlas/) where soil concentrations between 0.06 and 3.03 mg kg^−1^ for Cd and between 97 and 398 mg kg^−1^ for Zn are reported in the Spanish site of the cell. This considerable variability for Zn and Cd soil concentration was also reflected in this study where soils from Mauri and Freser had the same typically low Zn and Cd concentrations of non-metalliferous soils, while at Aneu two times higher concentrations of both metals were observed, along with somewhat higher Pb concentrations. These slightly enhanced metal levels are not due to industrial or mining activities in this zone but of geochemical origin as soils at Aneu are derived from shales.

This natural range in soil Zn and Cd concentrations is of special interest for the investigation of the mechanisms of metal accumulation and tolerance in the metal hyperaccumulating populations occurring on non-metalliferous soil. While intense research has been performed in *N. caerulescens* and clear population differences in the ability to accumulate and tolerate Zn and Cd have been described in this species (Escarré et al., 2000, 2013; Gonneau et al., 2014; Halimaa et al., 2014), *N. brachypetala* has been ignored for mechanistic studies. Hyperaccumulation of Zn, Cd, Pb, and/or Ni in *N. brachypetala* has occasionally been mentioned (Reeves and Brooks, 1983; Reeves et al., 2001; Koch and German, 2013); in all cases based on field or herbarium observations. However, the present data do not support accumulation of Pb by the examined plants. As far as we know, this is the first study not only proving the ability of *N. brachypetala* to hyperaccumulate both Zn and Cd under controlled-environmental conditions but also addressing population differences in Zn and Cd hyperaccumulation of this species in relation to differences in metal transporter gene expression. In fact, the shoot Zn and Cd concentrations of *N. brachypetala* plants (Figures 3B,D) growing in their natural habitat on soils with normal metal concentrations (Figures 3A,C) and soil pH values ranging from slightly acidic (Freser and Mauri) to near neutral (Aneu) did not hit the threshold concentrations of 10,000 mg kg^−1^ Zn and 100 mg kg^−1^ Cd usually considered for hyperaccumulator species (Baker and Brooks, 1989). Only plants from the Aneu population surpassed 3000 mg kg^−1^ Zn, the concentration more recently proposed as Zn hyperaccumulation threshold by several authors (van der Ent et al., 2013). However, these thresholds are typically reached or even widely surpassed in hyperaccumulator species growing on metalliferous soils. Here we report abnormally high shoot Cd and Zn concentrations in plants from normal soils, displaying bioaccumulation factors far above unity (Figure 4A).

This along with the results from the hydroponic experiments, where plants from all three *N. brachypetala* populations exceeded the shoot hyperaccumulation thresholds for Zn and Cd and accumulated higher shoot than root Cd and Zn concentrations, clearly demonstrates the hyperaccumulation character of *N. brachypetala* according to the more rational criteria recently established by van der Ent et al. (2013).

A further clear demonstration of the high efficiency of these *N. brachypetala* populations in hyperaccumulating Cd and Zn is the fact that when grown under the same hydroponic conditions these plants accumulated as high (Mauri for Cd) or even higher Cd and Zn shoot concentrations than *N. caerulescens* Ganges, an ecotype that is considered to be highly efficient in Cd and Zn hyperaccumulation (Ueno et al., 2011). Also root to shoot translocation factors for Zn and Cd were higher in the *N. brachypetala* populations than in *N. caerulescens* (Ganges) (Figure 4B). *Noccaea caerulescens* Ganges is highly adapted to metalliferous soils rich in Zn, and Cd (Lombi et al., 2000). Previous investigations have shown that *N. caerulescens* populations from non-metalliferous soils may accumulate higher Zn and Cd concentrations than those from metalliferous habitats (Escarré et al., 2000). This may be due to lower capacity for root vacuolar storage of metals leading to less exclusion from the transpiration stream. However, *N. caerulescens* populations from Zn-rich soil may exhibited higher Zn tolerance, than population from non-metalliferous; e.g., *N. caerulescens* plants from non-metalliferous soil but not those from the metalliferous substrate registered chlorosis when exposed to 50–100 μM of Zn (Assunção et al., 2003a). Despite the fact that the *N. brachypetala* populations described here developed on soils with less than 1 mg kg^−1^ Cd, all their offspring when hydroponically grown tolerated the accumulation of more than 200 μg g^−1^ Cd dry weight in their shoot tissues without any growth reduction, chlorophyll decrease, or influence on photosynthetic capacity. Root development was also not affected in *N. brachypetala* (data not shown). All *N. brachypetala* populations, excepting Freser, seem to tolerate exposure to 100 μM Zn as well as *N. caerulescens* (Ganges) (Figure 5). Nonetheless, further studies using higher external Zn concentrations are required to determine the Zn toxicity thresholds for the Aneu and Mauri populations. Freser was the most Zn sensitive population. Accumulation of more than 12,000 μg g^−1^ dry weight Zn in the shoots and more than 3000 μg g^−1^ Zn in the roots caused a significant decrease in biomass production in Freser.

Enhanced expression of metal transporter genes in combination with constitutively high amounts of both metal ligands with low to moderate binding strength such as histidine, nicotianamine, and organic acids, and antioxidants like phenolics and/or glutathione, are key factors for save metal transport to the shoots and metal compartmentation in the leaves in hyperaccumulating species (Tolrà et al., 1996; Freeman et al., 2005; Krämer, 2010; Schneider et al., 2013; Kozhevnikova et al., 2014). The expression of genes coding for metal transporters is regulated among others by the plant organ and its developmental stage, the metal concentration and the exposure time, and the availability of essential mineral nutrients (Küpper and Kochian, 2010).

Here we found differences in the expression of selected metal transporter genes amongst non-metallicolous populations of *N. brachypetala* and between *N. brachypetala* and *N. caerulescens* ecotype Ganges in response to the exposure to moderately high Cd and Zn concentrations. We focused our interest on *MTP1, HMA3*, and *HMA4*. The physiological and molecular basis of these genes are well-described for being related to Zn/Cd tolerance in different hyperaccumulator plants (Dräger et al., 2004; Ueno et al., 2011; Craciun et al., 2012). *NcHMA4* is expressed throughout the vascular system with highest expression in the crown and has been identified as a key player in both hyperaccumulation and hypertolerance of Zn and Cd (Hanikenne et al., 2008; Craciun et al., 2012). *HMA4* in roots seems responsible for xylem loading facilitating root to shoot metal transport (Hanikenne and Nouet, 2011). In *N. caerulescens* the transporter is localized in all leaf tissues (O'Lochlainn et al., 2011) and may help to transfer Zn from the xylem to the epidermal cells. Zinc compartmentation into the vacuole by the tonoplast transporter MTP1 may explain Zn tolerance in *A. halleri* (Krämer, 2005b). *MTP1* expression is also constitutively higher in the hyperaccumulating *N. caerulescens* than in the non-hyperaccumulating *Thlaspi arvense* (Assunção et al., 2001). Compartmentation of Cd into vacuoles is achieved by the tonoplast transporter *HMA3* and a higher expression of *NcHMA3* is required for Cd hypertolerance in the Cd-hyperaccumulating ecotype Ganges of *N. caerulescens* (Ueno et al., 2011).

Here we observed that the constitutive shoot expressions of *HMA4* and *HMA3* were substantially higher in *N. caerulescens* Ganges than in *N. brachypetala* (Figure S3). This is not surprising as *N. caerulescens* ecotype Ganges is highly adapted to metalliferous soils heavily polluted by Zn and Cd, while our *N. brachypetala* populations were from unpolluted sites. Noteworthy, Freser displayed higher root expression of *HMA4* under control conditions than Ganges (Figure S3). This is in accordance with the huge Zn shoot accumulation in this population. Faster translocation of metals from root to shoots in non-metallicolous than in metallicolous populations has previously been observed in both *N. caerulescens* and *A. halleri* accessions (Meerts and van Isacker, 1997; Bert et al., 2000; Assunção et al., 2001).

However, exposure to moderately high concentrations of Zn or Cd induced considerable differences in the expression patterns of the selected metal transporter genes among the non-metallicolous populations and between *N. brachypetala* and *N. caerulescens*. Different expression levels and the ecotype differences in metal uptake, transport, and tolerance in metal hyperaccumulating species like *N. caerulescens* or *Arabidopsis halleri* can be brought about by increased copy number of genes involved in Zn and Cd transport and compartmentation; but altered cis-regulation may be equally or even more important, at least in case of *HMA4*, which is demonstrated by studies with individual endogenous *N. caeruelescens* promoters (O'Lochlainn et al., 2011; Iqbal et al., 2013), and confirmed by “selection footprint analysis” (Hanikenne et al., 2013).

The differences in the relative expression of the three metal transporter genes *MTP1, HMA3*, and *HMA4* observed here, support the view of differences in the management of excess Zn and Cd amongst the studied species and populations (Figure 6). In *N. caerulescens* Ganges root vacuolar storage of Zn and Cd was apparently favored by up-regulation of *MTP1* and *HMA3* in the roots while the constitutively high shoot expression (Figure S3) did not change under the relatively mild stress conditions of this study (Figure 6). Amongst the *N. brachypetala* populations the high constitutive expression of *HMA4* in roots of Freser indicates high xylem loading; this high root expression level is maintained under Cd exposure and considerably enhanced in the shoots. In fact, Freser accumulated and tolerated the highest Cd shoot and root concentrations (Figure 4). Contrastingly, this population was rather sensitive to Zn (Figure 5). So the strong up-regulation of the Zn vacuolar transporter *MTP1* observed in the roots of Freser exposed to excess Zn for 1 week (Figure 6) did not lead to an efficient detoxification and restriction of Zn transport to the shoots in the longer term, as shown by the tissue concentration values determined after 4 weeks of exposure. In contrast, Mauri was Zn tolerant as indicated by the Zn-induced growth enhancement (Figure 5). Opposed to the other specimens, Mauri maintained or even reduced the root expression of *HMA3, HMA4*, and *MTP1* and exhibited the highest up-regulation of shoot *HMA4* under both Zn and Cd exposure (Figure 6). Zinc-induced changes in expression patterns in Aneu were similar to those found in Ganges. However, Cd supply caused a strong up-regulation of *HMA3* and *HMA4* in roots and of *MTP1* in shoots, while expression of *MTP1* was significantly reduced in the roots. These changes may favor Cd storage in the roots and Zn transport to the shoots under Cd stress. Taken together our results suggest differences amongst the hyperaccumulating *N. brachypetala* populations in the strategies for handling enhanced Cd and Zn availability: restriction of Cd uptake and enhanced Cd and Zn xylem loading in Mauri with Zn-induced growth stimulation; activation of root vacuolar storage of excess Zn, but insufficient for both restriction of shoot Zn accumulation and avoidance of Zn toxicity in Freser; in Aneu high Zn accumulation in both roots and shoots, high root vacuolar storage of Cd, and decreased root Zn vacuolar storage under Cd exposure favoring Zn translocation to the shoot.

*Noccaea brachypetala* was clearly identified based on morphological traits, while usual specific genetic markers failed to differentiate the three *Noccaea* species reported in the eastern Pyrenees. This study confirms for the first time the Zn and Cd hyperaccumulation character of *N. brachypetala* both in the field and in hydroponics according to criteria by van der Ent et al. (2013). Different expression patterns of metal transporter genes in response to Zn and Cd supply indicate different strategies for handling excess metal ions.

Knowledge about the genetic and molecular mechanisms of heavy metal transport in hyperaccumulators is of fundamental interest for the development of biotechnological tools for phytoremediation of metal contaminated soils. To date, most studies have focused only on two model plants, *N. caerulescens* and *Arabidopsis halleri* while close relatives like *N. brachypetala* have deserved little attention. Here we show for the first time that *N. brachypetala* is a Cd and Zn hyperaccumulator from non-metallipherous soil that responds to metal exposure by huge activation of metal transporter genes tolerating large concentrations of Zn and Cd in their leaves. The fact that these populations of *N. brachypetala* develop in the Pyrenees at the southern limit of distribution of *Noccaea* sp. may provide valuable additional genetic information not only regarding the genetic basis of metal accumulation and tolerance but also of adaptation to the distinctive climatic factors, especially summer heat and drought that will be of relevance for phytoremediation in Mediterranean areas.

## Author contributions

SM contributed to the experimental design, field collected, and lab processed samples, data elaboration, statistics, manuscript writing, and revision. BG contributed to field collected and lab processed samples, data elaboration, manuscript writing, and revision. LS and JL contributed to the data elaboration, statistics, and manuscript writing, and revision. CC and CP contributed to the experimental design, discussion, manuscript writing, and revision. All authors read and approved the manuscript.

### Conflict of interest statement

The authors declare that the research was conducted in the absence of any commercial or financial relationships that could be construed as a potential conflict of interest.

## Supplementary material

Figure S1 **Assay to test the ***N. caerulescens*** primers on ***N. brachypetala*****. Agarose gels for the amplicons generated by the HMA4 **(A)**, HMA3 **(B)** and MTP1 **(C)** primers. Alignments of the purified and sequenced amplicons for HMA4 **(D)**, HMA3 **(E)**, and MTP1 **(F)** regions. Gray boxes indicate the primer regions. Nc (G) is *N. caerulescens* ecotype Ganges, and Nb (A, F, and M) are *N. brachypetala* populations from Aneu, Freser, and Mauri, respectively.

Figure S2 **The 50% majority-rule consensus tree obtained from the Bayesian analysis of the combined ITS and cpDNA dataset indicating supported clades (***PP*** > 0.95)**. Numbers indicate the posterior probabilities (PP). Country names are given as the ISO standard. AV, seeds purchased from B&T World Seeds, Aigues-Vives, France. ^*^Indicates individuals from Herbarium BC. ^**^Indicates individuals from Herbarium BCN.

Figure S3 **Relative expression analysis of the three metal transporters according to the ΔCt method**. The expression is normalized by tubulin gene as a reference gene. Plants were grown on a hydroponic system for 1 week under metal treatment of 1.5 μM CdCl_2_ or 100 μM ZnSO_4_, control plants were non-exposed to Cd or with 2 μM Zn. Shoot markers expression is represented on the top part of the graphic and root markers expression on the bottom part.

Table S1 **Studied plant material from the herbaria of the Botanical Institute of Barcelona (BC) and the University of Barcelona (BCN)**.

Table S2 **Genbank accession numbers for the ITS1-ITS4, trnL-trnF, rpl32-trnL^(***UAG***)^ and trnQ- 5′rps16 regions**.

Table S3 **Primers used in the experiments**.

Table S4 **Numerical values of the morphometric multivariate analysis based on the correlation matrix**.
