# Supplementary figures and images for: Characterization of Zinc and Cadmium Hyperaccumulation in Three Noccaea (Brassicaceae) Populations from Non-metalliferous Sites in the Eastern Pyrenees
Source: Front Plant Sci. 2016 Feb 9;7:128. doi: 10.3389/fpls.2016.00128 (PMC4746256; doi:10.3389/fpls.2016.00128)

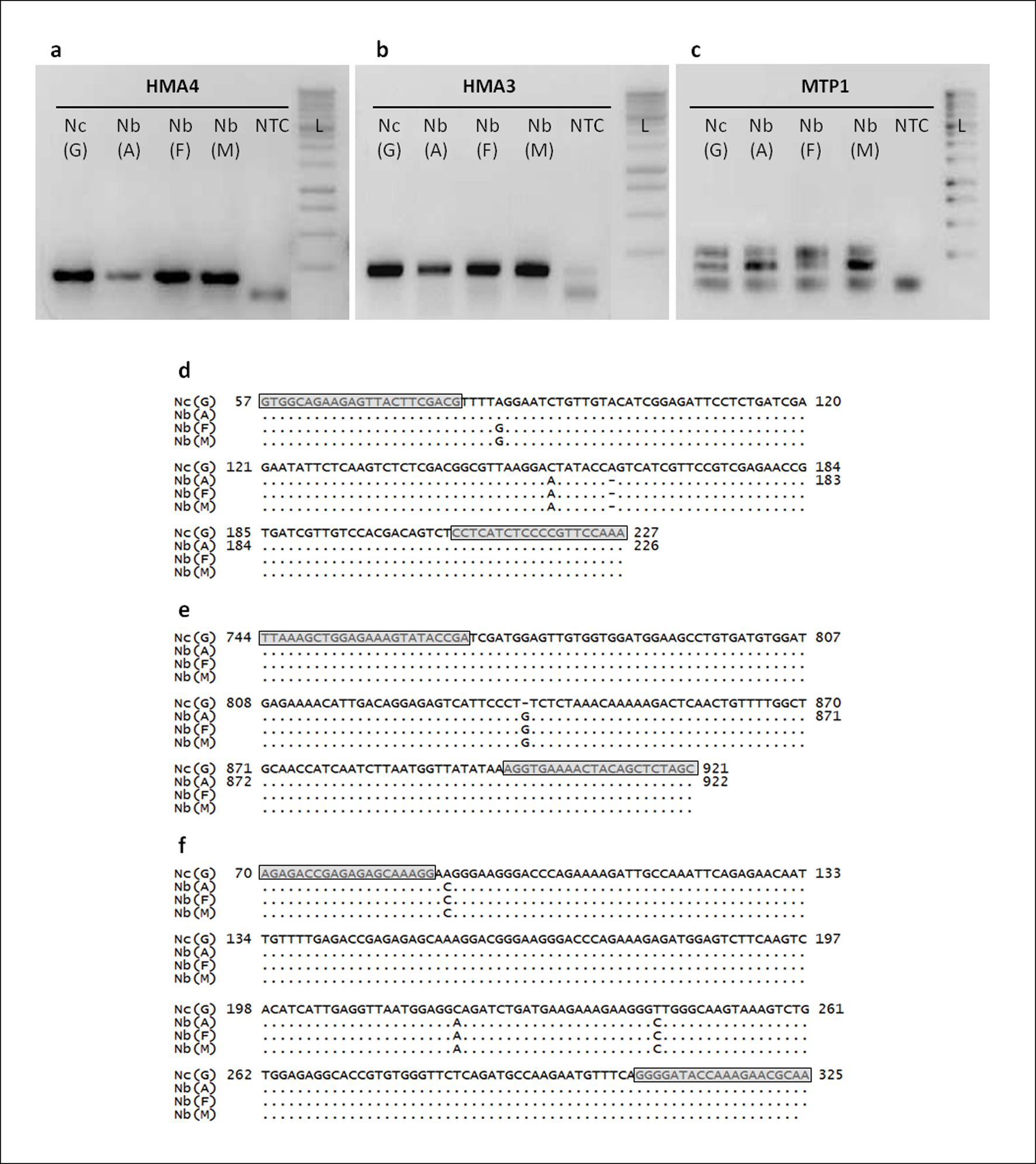

Supplement: Figure S1 — Assay to test the N. caerulescens primers on N. brachypetala. Agarose gels for the amplicons generated by the HMA4 (A), HMA3 (B) and MTP1 (C) primers. Alignments of the purified and sequenced amplicons for HMA4 (D), HMA3 (E), and MTP1 (F) regions. Gray boxes indicate the primer regions. Nc (G) is N. caerulescens ecotype Ganges, and Nb (A, F, and M) are N. brachypetala populations from Aneu, Freser, and Mauri, respectively. [file Image1.TIF]

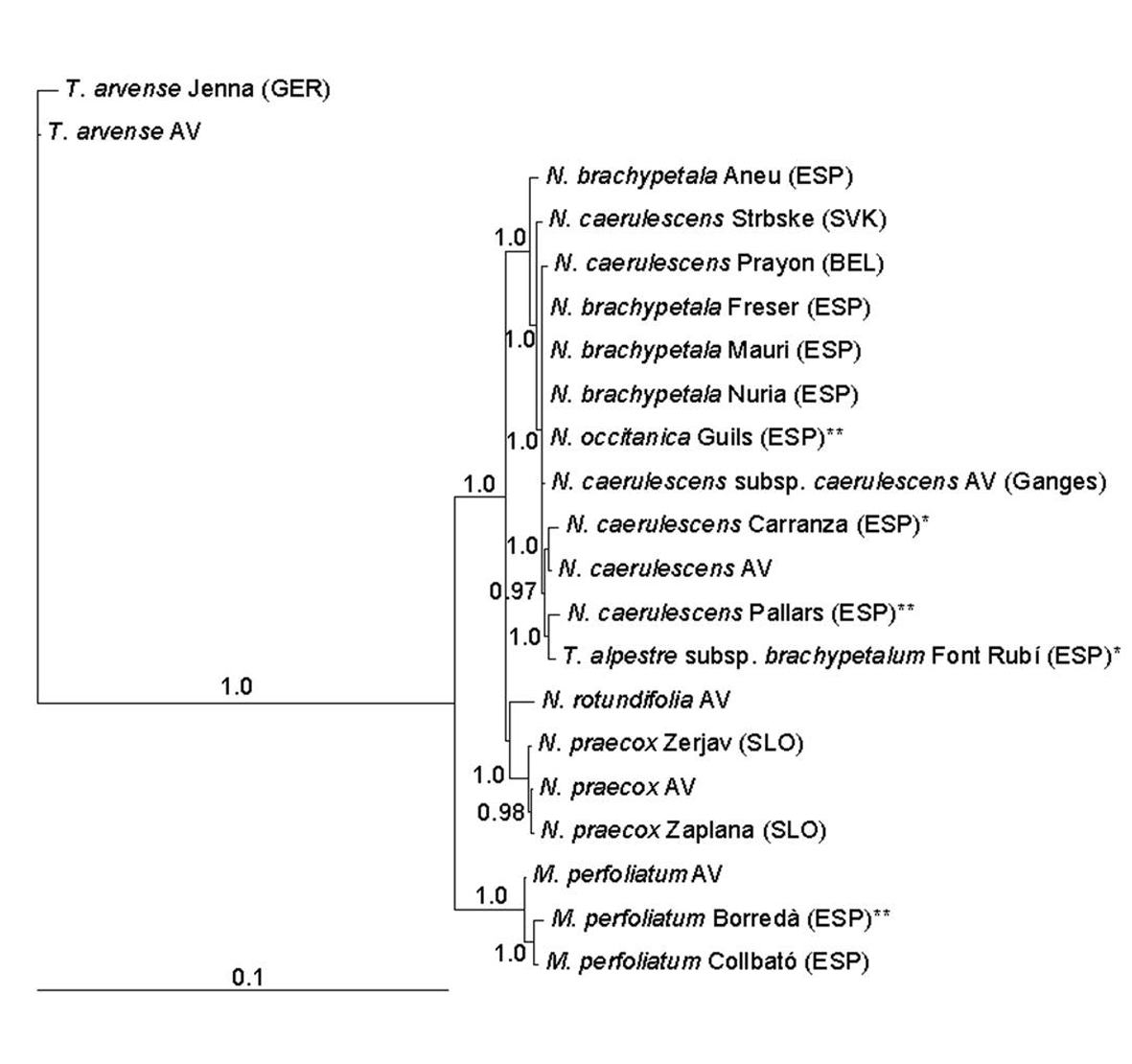

Supplement: Figure S2 — The 50% majority-rule consensus tree obtained from the Bayesian analysis of the combined ITS and cpDNA dataset indicating supported clades (PP > 0.95). Numbers indicate the posterior probabilities (PP). Country names are given as the ISO standard. AV, seeds purchased from B&T World Seeds, Aigues-Vives, France. *Indicates individuals from Herbarium BC. **Indicates individuals from Herbarium BCN. [file Image2.TIF]

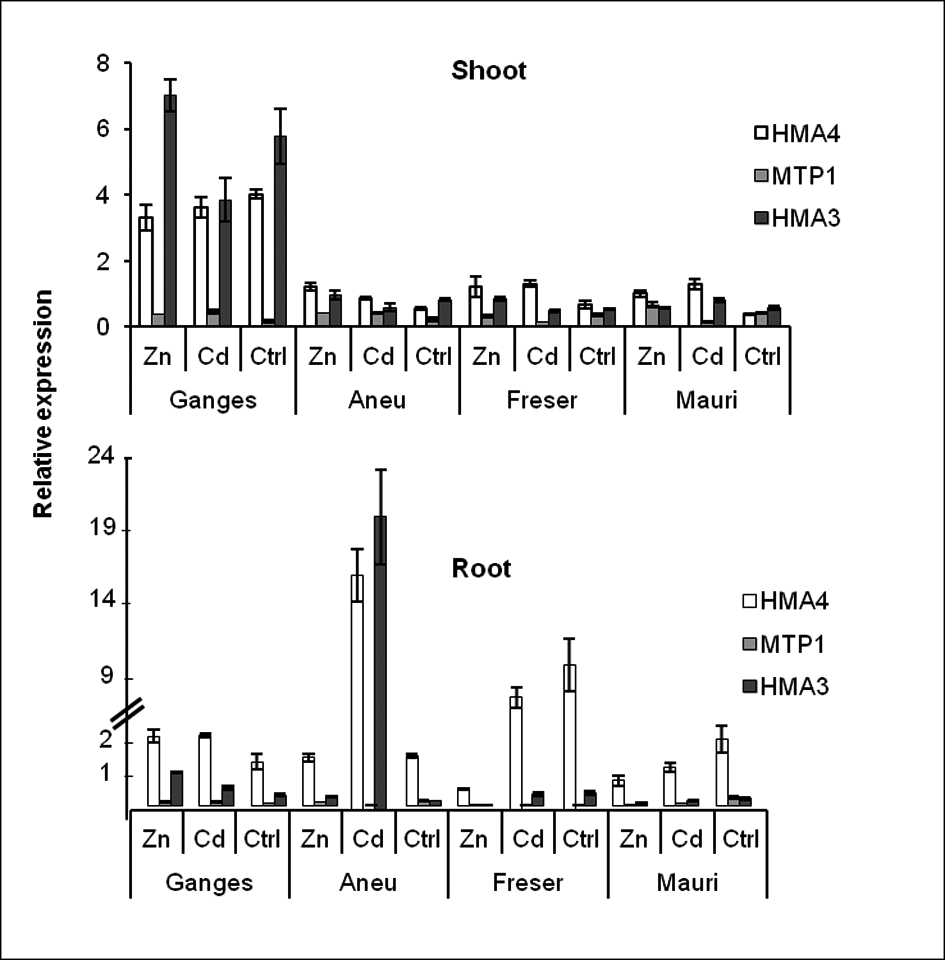

Supplement: Figure S3 — Relative expression analysis of the three metal transporters according to the ΔCt method. The expression is normalized by tubulin gene as a reference gene. Plants were grown on a hydroponic system for 1 week under metal treatment of 1.5 μM CdCl2 or 100 μM ZnSO4, control plants were non-exposed to Cd or with 2 μM Zn. Shoot markers expression is represented on the top part of the graphic and root markers expression on the bottom part. [file Image3.TIF]
