# Supplementary material for: Characterization of Zinc and Cadmium Hyperaccumulation in Three Noccaea (Brassicaceae) Populations from Non-metalliferous Sites in the Eastern Pyrenees
Source: Front Plant Sci. 2016 Feb 9;7:128. doi: 10.3389/fpls.2016.00128 (PMC4746256; doi:10.3389/fpls.2016.00128)
Supplement: Table S1 — Studied plant material from the herbaria of the Botanical Institute of Barcelona (BC) and the University of Barcelona (BCN). [file Table1.DOCX]

| **Studied plant material** |
| --- |
| *Noccaea brachypetala*: |
| **France**: Aveyron, La Lerezou, 800 m, 5 June 1892, H. Coste[?] (BC 856173 sub *Th. brachypetalum*); Pyrénees Orientales, Cerdagne, 1600 m, F. Sennen, 1897 (BC 856167); Font Romeu, Cerdagne, 1850 m, 30 July 1926, F. Sennen s.n. (BC 85616964, sub *Th. brachypetalum*); Estavar, 1270 m, 24 June 1927, F. Sennen s.n. (BC 856169, sub *Th. brachypetala*); Val d’Eyne, 1900 m, Aug[?] 1929, F. Sennen s.n. (BC 856163, sub *Th. brachypetalum*); Gorges de Llo, 1900 m, 17 Sept 1929, F. Sennen s.n. (BC 856172, sub *Th. brachypetala*). |
| **Spain**: Camprodón, 1875, R. de Bolos (BC 145051); Prados inmediatos al Santuario de Núria, 19 July 1880, Masferrer (BC 89200); La Rebuira, Areo, 1800 m; 19 July 1912, Font Quer (BC 5301); La Molinassa, Areo, 2000 m; 23 July 1912, Font Quer (BC 5298); Nuria, 2000 m, 1 Aug 1914, F. Sennen s.n. (BC 856172, sub *Th. brachypetalum*); Pyr.Centr. Espot, Ribera de S. Maurici, 1650 m, 12 July 1934, W. Rothmaler (BC 92174); Hospital de Viella, 8 Aug 1954, A. Bolos (BC 127893, sub *Th. alpestre*); Vall de Ribes, Núria, pr. Turó de la Creu, 2000 m; 9 July 1965, J. Vigo (BC 596342); Vall de Ribes, Gorges de Núria, 1900 m; 7 July 1968, J. Vigo & A. Anglada (BC 601514); Vallferrerra, Areu, 1400 m, 8 June 1975, J. Farreny (BC 620984); Nuria, prats, s.d., R. de Bolos (BC 113098); Prop de la Font Rubi, Alta Garrotxa, DG512863, 1025 m, s.d., P. Barnola (BC 868465, sub *Th. alpestre* subsp. *brachypetalum*) |
| *Noccaea caerulescens* subsp*. caerulescens* |
| **Spain**: Monte de la Regaliza, supra Leitoniegos,1700 m, 25 July 1935, Font Quer & Rothmaler (BC 650471); Mata de València, Pallars Sobirà, CH3922, 1600 m, prats, marges forestals, 15 May 2006, J.M. Ninot (BCN 59439, sub *Th. caerulescens* subsp. *caerulescens*); Son del Pi, Alt Aneu, Palomera, CH4118, 1890 m, Vizcaya, Carranza, Peña del Morro, 30TVN9189, 550 m, 11 May 2005, J. Elorza et al. (BC 917721); Prat, clariana de bosc, 1 June 2007, E. Carrillo & J.M. Ninot (BCN 59440, sub *Th. caerulescens* subsp*. caerulescens*); Baixa Cerdanya, Alp, torrent de Saltèguet, 31TDG1889, 1793 m, pradells secs entre el bosc de pi negre, 13 May 2007, A. Morell & J. Nuet NBJ0-046 (BCN 63869, sub *Th. alpestre* subsp. *alpestre*). |
| *Noccaea occitanica* |
| **France**: Cerdagne, Llivia, pâturages de la Néguilla, 1270 m, 24 June 1927, F. Sennen Pl. Espagne 6039 (BC856359, sub *Th. tallonis*); Llivia et Estavar, à la Néguilla, 1270 m, May 1928, F.S. Rémy Pl. Espagne 6506 (BC 856295, sub *Th. tallonis*, BCN, s.n.); Cerdagne Estavar et Llivia, gorges de la Néguilla, 1270 m, 6 May 1929, F. Sennen Pl. Espagne 7011 (BC 856297, sub *Th. tallonis*). |
| **Spain**: Baixa Cerdanya, Fontanals de Cerdanya, Pla de les Forques, 31TDG1492, 1700 m, pineda de pi negre i pi roig, 30 June 2007, C. Coll & F. Vallhonrat CAC0-004 (BCN 61620, sub *Th. caerulescens* subsp. *occitanicum*); Baixa Cerdanya, Guils, Girona, refugi i pleta de les cases, 31TDH0493302495, 1796 m, herbassars en terreny silici, 27 June 2011, I. Soriano, C. Rota, R. Merlo, S. Massó, A. Galián & E. Ollé (BCN 90332, sub *Th. caerulescens*) |

**Table S1** Studied plant material from the herbaria of the Botanical Institute of Barcelona (BC) and the University of Barcelona (BCN)
