# Supplementary material for: Characterization of Zinc and Cadmium Hyperaccumulation in Three Noccaea (Brassicaceae) Populations from Non-metalliferous Sites in the Eastern Pyrenees
Source: Front Plant Sci. 2016 Feb 9;7:128. doi: 10.3389/fpls.2016.00128 (PMC4746256; doi:10.3389/fpls.2016.00128)
Supplement: Table S2 — Genbank accession numbers for the ITS1-ITS4, trnL-trnF, rpl32-trnL(UAG) and trnQ- 5′rps16 regions. [file Table2.DOCX]

| **Organism** | **Origin** | **Herbarium** | **ITS region** | **trnL region** | **rpl32 region** | **rps16 region** |
| --- | --- | --- | --- | --- | --- | --- |
| *Microthlaspi perfoliatum* | Purchased from B&T World Seeds | - | KT220637 | KT253348 | KT220658 | KT253370 |
| *M. perfoliatum* | Borredà (ESP) | BCN103124 | KT220639 | KT253350 | KT220660 | KT253372 |
| *M. perfoliatum* | Collbató (ESP) | Plant Physiology UAB collection | KT220638 | KT253349 | KT220659 | KT253371 |
| *Noccaea brachypetala* | Aneu (ESP) | PP UAB coll. | KT220631 | KT253342 | KT220653 | KT253364 |
| *N. brachypetala* | Freser (ESP) | PP UAB coll. | KT220632 | KT253343 | KT220654 | KT253365 |
| *N. brachypetala* | Mauri (ESP) | PP UAB coll. | KT220633 | KT253344 | KT220655 | KT253366 |
| *N. brachypetala* | Mauri2 (ESP) | PP UAB coll. | KT220634 | KT253345 | - | KT253367 |
| *N. brachypetala* | Nuria (ESP) | PP UAB coll. | KT220635 | KT253346 | KT220656 | KT253368 |
| *Noccaea caerulescens* | Carranza (ESP) | BC917721 | KT220622 | KT253333 | KT220644 | KT253355 |
| *N. caerulescens* | Pallars (ESP) | BCN59439 | KT220623 | KT253334 | KT220645 | KT253356 |
| *N. caerulescens* | Prayon (BEL) | PP UAB coll. | KT220621 | KT253332 | KT220643 | KT253354 |
| *N. caerulescens* | From B&T | - | KT220624 | KT253335 | KT220646 | KT253357 |
| *N. caerulescens* | Strbske (SVK) | PP UAB coll. | KT220625 | KT253336 | KT220647 | KT253358 |
| *N. caerulescens* subsp*. caerulescens* | From B&T | *-* | KT220626 | KT253337 | KT220648 | KT253359 |
| *Noccaea occitanica* | Guils (ESP) | BCN90332 | KT220636 | KT253347 | KT220657 | KT253369 |
| *Noccaea praecox* | From B&T | *-* | KT220627 | KT253338 | KT220649 | KT253360 |
| *N. praecox* | Zaplana (SLO) | PP UAB coll. | KT220629 | KT253340 | KT220651 | KT253362 |
| *N. praecox* | Zerjav (SLO) | PP UAB coll. | KT220628 | KT253339 | KT220650 | KT253361 |
| *Noccaea rotundifolia* | From B&T | *-* | KT220640 | KT253351 | KT220661 | KT253373 |
| *Thlaspi alpestre* subsp. *brachypetalum* | Font Rubí (ESP) | BC868465 | KT220630 | KT253341 | KT220652 | KT253363 |
| *Thlaspi arvense* | Jenna (GER) | PP UAB coll. | KT220619 | KT253330 | KT220641 | KT253352 |
| *T. arvense* | From B&T | - | KT220620 | KT253331 | KT220642 | KT253353 |

**Table S2** Genbank accession numbers for the ITS1-ITS4, trnL-trnF, rpl32-trnL^(UAG)^ and trnQ- 5’rps16 regions.
