# Supplementary material for: Characterization of Zinc and Cadmium Hyperaccumulation in Three Noccaea (Brassicaceae) Populations from Non-metalliferous Sites in the Eastern Pyrenees
Source: Front Plant Sci. 2016 Feb 9;7:128. doi: 10.3389/fpls.2016.00128 (PMC4746256; doi:10.3389/fpls.2016.00128)
Supplement: Table S3 — Primers used in the experiments. [file Table3.DOCX]

**Table S3** Primers used in the experiments.

| **Primers** | **Sequence (5’-3’)** | **References** |
| --- | --- | --- |
| ITS1 | TCCGTAGGTGAACCTGCGG | White *et al*., 1990 |
| ITS4 | TCCTCCGCTTATTGATATGC |  |
| *trn*L (primer e) | GGTTCAAGTCCCTCTATCCC | Taberlet *et al*., 1991 |
| *trn*F (primer f) | ATTTGAACTGGTGACACGAG |  |
| rpL32F | CAGTTCCAAAAAAACGTACTTC | Shaw *et al*., 2007 |
| trnL^(UAG)^ | CTGCTTCCTAAGAGCAGCGT |  |
| trnQ^(UUG)^ | GCGTGGCCAAGGGTAAGGC | Shaw *et al*., 2007 |
| rpS16x1 | GTTGCTTTTACCACATCGTTT |  |
| qPCR-HMA3F | TTAAAGCTGGAGAAAGTATACCGA | Ueno *et al*., 2011 |
| qPCR-HMA3R | GCTAGAGCTGTAGTTTTCACCT |  |
| qPCR-NcHMA4F | GTGGCAGAAGAGTTACTTCGACG | Iqbal *et al*., 2013 |
| qPCR-NcHMA4R | TTTGGAACGGGGAGATGAGG |  |
| qPCR-NcMTP1F | AGAGACCGAGAGAGCAAAGG | Klein *et al.,* 2008 |
| qPCR-NcMTP1R | TTGCGTTCTTTGGTATCCCC |  |
| qPCR-TubulineF | CTACGCACCAGTCATCTCT | Wu *et al*., 2009 |
| qPCR-TubulineF | CGAGATCACCTCCTGGAACA |  |
