# Supplementary material for: Characterization of Zinc and Cadmium Hyperaccumulation in Three Noccaea (Brassicaceae) Populations from Non-metalliferous Sites in the Eastern Pyrenees
Source: Front Plant Sci. 2016 Feb 9;7:128. doi: 10.3389/fpls.2016.00128 (PMC4746256; doi:10.3389/fpls.2016.00128)
Supplement: Table S4 — Numerical values of the morphometric multivariate analysis based on the correlation matrix. [file Table4.DOCX]

| Morphometric studied characters | Principal Components | |
| --- | --- | --- |
|  | 1 | 2 |
| Stem W | 0,867 | 0,135 |
| Stem L | 0,679 | 0,616 |
| Inflor L | 0,503 | 0,533 |
| Inflor L/Stem L | -0,464 | -0,476 |
| Fruiting Pedicel L (max) | 0,446 | -0,119 |
| Style L | -0,782 | -0,126 |
| Notch L | 0,780 | -0,572 |
| Style L/Notch L | -0,839 | 0,372 |
| Fruit L | 0,857 | 0,208 |
| Fruit W | 0,535 | -0,751 |
| Fruit Wing W (at apex) | 0,887 | -0,353 |
| Fruit L/Fruit W | 0,251 | 0,900 |
| **% of explained variance** | | |
| PC1 | 47,295 |  |
| PC2 | 24,54 |  |
|  | 71,835 |  |

**Table S4** Numerical values of the morphometric multivariate analysis based on the correlation matrix
